# VALUING AND SUPPORTING THE INVISIBLE WORKFORCE OF FAMILY CAREGIVERS

**DOI:** 10.1093/geroni/igad104.0792

**Published:** 2023-12-21

**Authors:** Susan Reinhard

## Abstract

The 2023 update to the Valuing the Invaluable series estimates the economic value of unpaid, family caregiving in America was $600 billion in 2021. This estimate continues a 25-year trend of increasing economic value for family caregiving, which fills an essential role in the fractured LTSS system. This work also details the diversity of family caregivers and their care experiences, the challenges that caregivers face juggling care with work and child care, and the impact of direct care worker shortages on family caregivers. This symposium showcases the national and state estimates of the economic value of caregiving, details who caregivers are today, what challenges they face, and what policies and practices can support and recognize them and the vital care they provide. Presenter 1 shares an overview of the methodology used in this report and the national and state estimates of the economic value of caregiving. Presenter 2 discusses the increasing generational, racial and ethnic, and sexual identity and orientation diversity of family caregivers today and the challenges specific to different communities of caregivers. Presenter 3 highlights promising policies and practices to support caregivers and details recommendations for building on those promising policies and practices. This is a Family Caregiving Interest Group Sponsored Symposium.

